# Pathological Specimen—Extirpation of a Thyroid Gland Weighing Sixteen and One-halt Ounces—Recovery

**Published:** 1884-09-20

**Authors:** 


### PATHOLOGICAL SPECIMEN.—EXTIRPATION OF A THYROID GLAND WEIGHING SIXTEEN AND ONE-HALF OUNCES.—RECOVERY.

**At St. Louis Medical Society.**

DR. MUDD.—I have a specimen which is of interest. On the morning of June 7th at about 8 o'clock I was called to see a young man, aged 18, whom I found unconscious; he had stertorous, irregular breathing and a slow pulse. He had a very large thyroid gland. At two o'clock A. M. he arose to get a drink of water, did not obtain it and returned to the side of his couch, was spoken to by a bed-fellow, did not answer, but tumbled on the bed unconscious and remained so until I saw him at 8 o'clock A. M. I could feel, passing across the median line, the isthmus of the thyroid body, which was elevated, and the trachea was depressed on its upper margin. I made at once tracheotomy without an anæsthetic. I had only the ordinary tubes with me. I passed one of them in and found it did not relieve his breathing. I then passed the ordinary gum catheter down the trachea, two inches and a half perhaps, and the breathing became easier. I obtained a bulb sound and a metal catheter of large size, No. 13 perhaps, and passed the former through the stricture, which was a little below the border of the sternum or near the lower portion of the trachea, and I could feel distinctly the point at which the trachea was flattened by the pressure of the tumor. I bent the catheter and passed it below that point, whereupon he breathed quite freely and easily. He did not recover consciousness till ten hours after the operation, which was 5 o'clock P. M., when he became somewhat conscious; he was still dull, but could be aroused; his lips had regained their color and his circulation was very much better. His temperature rose to 101 or 102 on the second day. On June 10th at one o'clock, notwithstanding the temperature of 101 6-10 and a pulse of 120, it was deemed best to extirpate the thyroid gland. It extended downward under the sternum upon both sides, was full and rounded, and pointed backward, the right side being the larger; it was very prominent below on the left and somewhat higher up than on the right; upon either side it extended upward to the superior border of the thyroid cartilage and towards the angle of the jaw. The patient was anæsthetized by placing a rubber tube over the opening of the catheter, which projected from the neck, and to that was attached a reservoir of ether; he breathed the ether in that way and came very promptly under its influence. The incision was made in the median line, the isthmus .exposed, the muscles turned back, the gland enucleated upon the right side and then upon the left. There was less difficulty in the operation than was anticipated. I had feared that he would die on the table because of the extension of the tumor along the neck and behind the sternum, but it was rather easily enucleated and the vessels secured There was considerable shock and the hemorrhage was quite free, but the patient passed through the operation admirably. After the extirpation of the gland on the right side I turned it out; the attachment was so firm at the isthmus that before I was through on the left side, the right portion of the thyroid broke off and dropped upon the floor. The bleeding was free, but easily controlled; the adhesions were firmest at the site of its connection with the thyroid cartilage and the borders of the cricoid, and I had some difficulty in removing them; at the point of the superior and inferior thyroid they were easily removed. The point of greatest interest was the appearance of the trachea, which was exposed throughou talmost its entire extent: it was flattened so that the lateral was, perhaps, not more than one-third of its anterio-posterior diameter. The point of greatest flattening of the rings was near the lower portion of the tumor, as it presented behind the sternum. There was a question as to the ability of the trachea maintaining sufficient dilation if I removed the tube, so the tube was allowed to remain in and the breathing continued easy and free. The shock was greatest about 18 hours after the operation, when his pulse was imperceptible; he was also a little delirious, with irregular breathing, and it looked as if he would die; but he had an atropine injection, rallied promptly and made a good recovery. The last ligature was removed about eight days after the operation. The wound is practically well and he talks and breathes easily. In preparing for the operation I detached the sternal portion of the sterno-cleido muscle and turned it back, so as to afford more room in the lower portion of the neck where I expected most trouble. The disturbance of respiration was not occasioned by pressure on the recurrent laryngeal, but was entirely dependent upon mechanical compression of the trachea with consequent disturbance of respiratory movements of the glottis. The patient had suffered for three years wi h this tumor, which had grown somewhat rapidly during the last three months; it was quite firm in character, and for two or three weeks prior to the time when he became unconscious he slept badly; during the ten days preceding the operation he had had no fever and was so well that he was attending to business. He had been spending the evening with friends, went to bed at eleven o'clock and, probably, by some unusual position of the head in falling on the bed became unconscious, the breathing was interrupted by a sudden bend of, with tumor pressure upon the trachea; he was certainly distressed and not breathing easily when he aroused himself to get water, and his companions thought he would die almost immediately; he looked like he was dying, but rallied somewhat, never, however, becoming conscious, and was nearly dead when I first saw him. The specimen is interesting because of its size and comparative rarity; it weighed a little over 16½ ounces. There is a point of importance in connection with this case: after the tracheotomy the tube was removed several times for cleansing; we had no double tube, and it became occasionally occluded. At these times the trachea collapsed and he would have suffocated if he had been left only a few minutes without a tube; hence, I obtained two tubes and when I removed one introduced the other. The operation was undertaken simply because there was no other course to pursue. The man was 18 years of age and was otherwise healthy. He was born in Cincinnati and left there about 18 months since for this city. Since then he has been here actively engaged in an office.

*Dr. Pollak.*—What was his occupation?

*Dr. Mudd.*—He was clerk in an insurance office.

*Dr. Hughes.*—Was there any disturbance of the respiratory movements except at the time of his paroxysm, or any irregular accelerated action of the heart?

*Dr. Mudd.*—I never saw him till he was unconscious; there had been disturbance of the respiratory movements, as shown by the changed relation of the ribs to the costal cartilages; they were somewhat flattened and depressed, as if from labored respiration.

*Dr. Hughes.*—A number of cases of disturbed respiration due to nerve pressure have been reported, and it would be interesting to know if this was the condition in this case.

*Dr. Pollak.*—How do you account for the patulency of the trachea?

*Dr. Mudd.*—I think the soft tissues about it support it somewhat; there was no pressure, probably, upon the trachea after the removal of the tumor and it is now sustained by granulations. I think, probably, the soft parts will help to keep it in position and pervious.

*Dr. Pollak.*—How long did the operation last?

*Dr. Mudd.*—About an hour.

*Dr. Pollak.*—Did you do it under antiseptic treatment?

*Dr. Mudd.*—No, sir; we used clean sponges but did not use the spray. The wound was washed with a corrosive sublimate solution and dressed with an antiseptic pad.—*St. Louis Medical and Surgical Journal.*

